# Different Endurance Exercise Modalities, Different Affective Response: A Within-Subject Study

**DOI:** 10.3389/fpsyg.2021.686661

**Published:** 2021-08-13

**Authors:** Katja Dierkes, Felipe Mattioni Maturana, Inka Rösel, Peter Martus, Andreas M. Nieß, Ansgar Thiel, Gorden Sudeck

**Affiliations:** ^1^Faculty of Economic and Social Sciences, Institute of Sports Science, University of Tübingen, Tübingen, Germany; ^2^Interfaculty Research Institute for Sport and Physical Activity, University of Tübingen, Tübingen, Germany; ^3^Department of Sports Medicine, University Hospital of Tübingen, Tübingen, Germany; ^4^Faculty of Medicine, Institute for Clinical Epidemiology and Applied Biometry, University of Tübingen, Tübingen, Germany

**Keywords:** affect, physical activity, exercise, dual-mode theory, variability, within-subject, cognitive factors, interoceptive cues

## Abstract

Affect experienced during an exercise session is supposed to predict future exercise behavior. However, empirical evidence reveals high variability in affective response to different exercise modalities. Thus, the purpose of the present study was to compare acute affective response and its variation during three different endurance exercise modalities: (a) moderate-intensity continuous exercise (MICE), (b) vigorous-intensity continuous exercise (VICE), and (c) high-intensity interval exercise (HIIE). Using the dual-mode theory as a theoretical framework, cognitive and interoceptive factors were considered as potential predictors of in-task affective response. In a within-subject design, 40 insufficiently active healthy participants (aged from 20 to 40 years) attended three sessions per exercise modality on a cycle ergometer. Affective valence (measured by the Feeling Scale), two cognitive factors (perceived competence and awareness of interoceptive cues), and one interoceptive factor (heart rate) were assessed before, during, and after each exercise session. Mixed models with three levels (*subject, exercise session*, and *time point*) revealed more positive affective valence during MICE compared with VICE (*p* < 0.001) and HIIE (*p* < 0.01), while there was no significant difference between the latter two. Levene's test results showed the highest variability of in-task affective valence during VICE (*p*s < 0.01). Regarding the course across the session, MICE was associated with a constant slight increase in affective valence from pre- to post-exercise (*p* < 0.05), whereas VICE and HIIE caused a decline in pleasure, followed by an affective rebound immediately after exercise termination (*p*s < 0.01). The highest importance of cognitive and interoceptive factors for in-task affective valence was observed in VICE (*p*s < 0.05). The current findings provide support for the tenets of the dual-mode theory, however, indicating that there may be differences in the affect-intensity relationship between continuous and interval exercise. In conclusion, the study results concerning previously insufficiently active individuals extend the knowledge of how exercise can positively shape affective well-being depending on exercise modality and psychophysiological influences. This knowledge enables public health practitioners to design more individualized activity recommendations, thereby improving the subjective experience of exercise.

## Introduction

In the context of theory-based interventions to enhance adoption and maintenance of exercise programs, affective response during an exercise session has been shown to predict future exercise behavior (Ekkekakis and Dafermos, 2012; Williams et al., 2012; Rhodes and Kates, 2015). However, substantial interindividual response variability has been demonstrated (Ekkekakis et al., 2011) and exercise characteristics (e.g., intensity, modality) seem to have a decisive influence (Stork et al., 2017). Affective response can be viewed as an umbrella term for numerous interrelated constructs, while current literature has highlighted *core affective valence* as the crucial component (Ekkekakis et al., 2020; Stevens et al., 2020), which is primitive and limited to basic appraisals of pleasure and displeasure (Russell and Barrett, 1999).

To date, research has not sufficiently clarified which specific factors influence the affective response and its variability during various exercise modalities. In order to prevent affective experiences that could obviate exercise adherence, this knowledge is of particular relevance. Thus, the purpose of the present study was to investigate affective response during three endurance exercise modalities considering different exercise intensities among insufficiently active adults. Providing explanations for variability in individuals' affective response will give insight into how exercise can be structured to achieve more positive affective states (i.e., how to make exercise more pleasant).

### Affective Response Depending on Exercise Intensity

A broader conceptual framework that encompasses exercise intensity-dependent patterns of interindividual variability in affective response is the dual-mode theory (DMT; Ekkekakis, 2003, 2005). On the grounds of evolutionary arguments and adaptational implications, differences in affective response to exercise are explained by the continuous interplay between two general factors: (a) cognitive parameters (e.g., perceived competence, awareness of interoceptive cues), and (b) interoceptive stimuli (i.e., those emerging from the body such as increased heart rate and ventilation). The relative contribution of these two factors is hypothesized to shift systematically as a function of exercise intensity and the corresponding metabolic requirements (Ekkekakis and Acevedo, 2006; Ekkekakis, 2009). First, for exercise within the *moderate-intensity domain* (below the first ventilatory or lactate threshold [VT1/LT1]), it is assumed that affective responses are interindividually relatively homogeneous and mainly positive, with a small to moderate influence of cognitive factors (Ekkekakis et al., 2005a). Due to the low metabolic requirements, such intensities can be maintained over a long period of time and do not pose a threat to the homeostasis of the organism, consequently resulting in pleasant sensations (e.g., feelings of warmth; Ekkekakis and Acevedo, 2006). Second, in the *heavy-intensity domain* (extending from VT1/LT1 to the maximal lactate steady state [MLSS]), the highest variability of affective response is postulated, with some individuals reporting increases and others decreases in pleasure (Ekkekakis et al., 2005a). Rising blood lactate concentration and associated physiological processes to maintain the activity represent a challenge to the adaptive capacity of the body, without implying any concrete utility or danger. Given the ambiguous adaptational implications of exercise within this intensity range, cognitive factors are assumed to determine the affective response (Ekkekakis and Acevedo, 2006). Third, exercise within the *severe-intensity domain* (extending from MLSS to the level of maximal exercise capacity; above the VT2/LT2) is supposed to elicit again more interindividually homogeneous affective responses, but this time in the form of a decrease in pleasure (Ekkekakis et al., 2005a). Activities in this intensity range are based on limited energetic resources of the anaerobic metabolism and preclude the maintenance of a physiological steady state. A strong interoceptive influence (e.g., heart rate, blood lactate) is assumed here, which signals the approaching state of exhaustion to the body (i.e., adaptational risk) and provokes the timely termination of the exercise in order to prevent damage to the systems. Interoceptive stimuli thus act as a kind of protective mechanism, causing unpleasant sensations during exercise in this critical physiological range (Ekkekakis and Acevedo, 2006).

Exercise intensity-dependent patterns of affective response that are apparent during exercise tend to dissipate rather rapidly as soon as the activity is terminated (Ekkekakis et al., 2011). Accordingly, Backhouse et al. (2007) suggested that the “feel-better” effect of exercise may be an artifact of just measuring pre-post affective response, failing to account for affective fluctuations across an exercise session. While exercising within the moderate domain (i.e., intensities below VT1/LT1) should be accompanied by a constant or slightly increasing trend in affective valence, exercising at intensities at or above VT1/LT1 is supposed to cause a decline in pleasure, followed by a robust positive “affective rebound” once exercise is terminated. That is, a uniform shift toward pleasure after exercising in the heavy- or severe-intensity domain is predicted by DMT (Ekkekakis et al., 2005a). As a result, exercisers are supposed to return to or even exceed pre-exercise feeling states (Ekkekakis et al., 2011). Within the literature on DMT, this assumption is based on the “affect contrast” phenomenon described by Solomon (1980). Here, the opposing trend in the pattern of affective response is attributed to an adaptive benefit of terminating aversive stimuli and a concomitant restoration of homeostasis. As a result of an increased production of neuromodulators (e.g., endorphins, dopamine, serotonin), an increase in pleasure after intense exercise is assumed (Solomon, 1991; Basso and Suzuki, 2017).

### Comparing Different Endurance Exercise Modalities: Empirical Evidence

Research on DMT provides broad support within the context of continuous exercise regarding the valence and variability of affective responses (e.g., Ekkekakis et al., 2011, 2018; Oliveira et al., 2015). Thus, empirical findings show less positive and more variable responses during vigorous-intensity continuous exercise (VICE; i.e., heavy domain) in comparison with moderate-intensity continuous exercise (MICE; i.e., moderate domain). In contrast, affective response during interval exercise is less understood. Recently, high-intensity interval exercise (HIIE) has been gaining attention in the public health area as a time-efficient, less monotonous exercise strategy to improve cardiorespiratory and metabolic health (e.g., Batacan et al., 2017; Mattioni Maturana et al., 2021a). HIIE is characterized by relatively brief, repeated bouts at maximal or near-maximal effort, interspersed with recovery periods of low intensity or complete rest (Gillen and Gibala, 2014). The numerous benefits of HIIE on physiological outcomes are well-documented; however, concerns have been raised about the likelihood of HIIE (i.e., exercise in the severe-intensity domain) evoking a high degree of displeasure (Decker and Ekkekakis, 2017).

In recent years, an increasing number of studies have investigated differences in affective response between HIIE and continuous exercise, so review-based evidence is now available. Niven et al. (2020) performed a meta-analytic synthesis of the current research (HIIE vs. MICE: 15 studies; HIIE vs. VICE: 7 studies) and concluded that, while HIIE is associated with more negatively valenced affective responses during exercise when compared with MICE, there is no difference in in-task affective valence between HIIE and VICE. Importantly, a large degree of heterogeneity was evident in both comparisons. A detailed discussion on the apparent inconsistency regarding affective response to interval in comparison with continuous exercise can be found elsewhere (Decker and Ekkekakis, 2017).

Regarding the affective-rebound effect, the empirical data confirmed the assumptions of DMT for exercise at or above the VT1/LT1. Resembling the numerous findings on continuous exercise in the heavy domain (i.e., VICE; e.g., Ekkekakis et al., 2008, 2011), the affective valence decreased consistently during HIIE. However, as assumed, this decline in pleasure was followed by an affective rebound immediately after exercise termination or in the post-exercise period, returning to or even exceeding baseline values (Oliveira et al., 2013; Jung et al., 2014; Niven et al., 2018; Stork et al., 2018; Alicea et al., 2020; Box et al., 2020).

To date, not much is known about exercise modality-dependent factors of affective valence and its variation. However, among other cognitive factors, concepts relating to perceptions of ability (e.g., perceived competence) and attentional focus (e.g., awareness of interoceptive cues) have been shown to account for variation in the valenced (pleasure–displeasure) response to continuous exercise within the moderate and especially within the heavy domain (Rose and Parfitt, 2007; Ekkekakis et al., 2011). Recently, a narrative review found perceived competence to be a consistent explanatory variable for affective valence (Bourke et al., 2020). This is in line with the important role attributed to competency-related characteristics in prevalent theories of behavior change (e.g., Self-Determination Theory; Deci and Ryan, 1985, Social Learning Theory; Bandura, 1977). In contrast, mixed results were found for the concept of attentional focus (Bourke et al., 2020). Rose and Parfitt (2010) illuminate such mixed results in exercising around VT1, showing that some individuals interpret interoceptive cues positively and others negatively. Thus, the directional influence of the awareness of interoceptive cues on affective valence seems to be highly variable, depending on how the cues are interpreted (i.e., individual's cognitive appraisals of the stimuli). Still less studied is the negative influence of interoceptive stimuli (e.g., heart rate) on affective response in the severe domain postulated by DMT, although recent evidence supports the link between affective valence and homeostatic perturbations (Hartman et al., 2019).

Despite emerging trends, the comparison of affective response between continuous and interval-based exercise is still insufficiently elucidated. Due to the large number of existing endurance exercise modalities, barely any reliable predications can be made. In addition, the question arises as to which psychophysiological factors are relevant in which exercise modality, and to what extent these are related to the variability of affective response.

### Study Rationale

This study aimed to investigate acute affective response to different endurance exercise modalities in previously insufficiently active individuals. Covering the exercise intensity domains described within DMT, the following three exercise modalities were compared: (a) MICE (i.e., moderate domain), (b) VICE (i.e., heavy domain), and (c) HIIE (i.e., severe domain).

Within this approach, we first wanted to explore differences in *valence and variation of in-task affective response* among the three different exercise modalities. With respect to the outlined theoretical arguments regarding continuous exercise (MICE and VICE) and review-based evidence on HIIE (in comparison with MICE and VICE), we hypothesized that MICE would result in more positive in-task affective valence compared with VICE and HIIE, while there would be similar affective responses between the latter two (MICE > VICE, HIIE [Hypothesis H1a]). We moreover assumed that, in line with the DMT postulate, VICE would be associated with a higher variability of in-task affective valence in comparison with MICE (VICE > MICE [Hypothesis H1b]).

Second, we examined whether the *course of affective response across the session* differs depending on the exercise modality. Considering the assumptions of DMT and the high consistency of research findings, we hypothesized that MICE would be on average accompanied by a constant or slightly increasing trend in affective valence from pre- to post-exercise, while VICE and HIIE (i.e., intensities above the VT1/LT1) would cause a decline in pleasure, followed by a positive affective rebound immediately after exercise termination (Hypothesis H2).

Lastly, we aimed to identify *psychophysiological predictors of in-task affective response*. As cognitive factors, we first considered perceived competence (PC) as an already highlighted important explanatory variable for affective valence. Second, within the concept of attentional focus, we examined the awareness of interoceptive cues (AOI) as a less consistent variable. Based on the DMT postulate and previous studies, we hypothesized that cognitive factors (PC and AOI) would be more strongly associated with in-task affective valence in VICE than in MICE (VICE > MICE; Hypothesis H3). Since evidence for interval exercise is particularly scarce in this regard, no directional hypothesis for HIIE was formulated. Third, we examined the association of heart rate (HR), the interoceptive factor, with in-task affective valence on an exploratory basis.

## Materials and Methods

### Study Design

**Figure 1:**
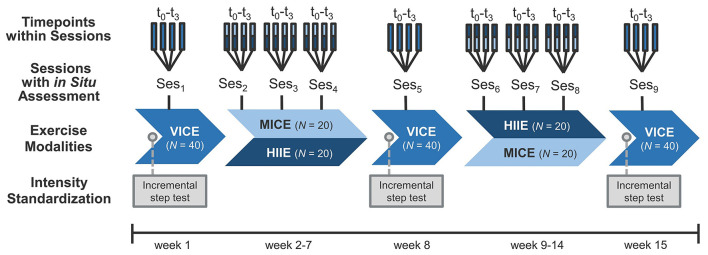
Overview of the within-subject design. *In situ* assessments were conducted at 4 time points (t_0_-t_3_) within 3 exercise sessions per exercise modality (VICE, vigorous-intensity continuous exercise; MICE, moderate-intensity continuous exercise; HIIE, high-intensity interval exercise; Ses_1−9_), resulting in a total number of 36 observations per participant (*N* = 40). In laboratory visits at weeks 1, 8, and 15, exercise intensities were standardized using an incremental step test with lactate diagnostics and spiroergometry.

This study was part of a larger research initiative entitled “Individual Response to Physical Activity—A Transdisciplinary Approach” (iReAct; Thiel et al., 2020). The iReAct project is an interdisciplinary research network, investigating individual physiological, affective, and cognitive responses based on a randomized, two-period sequential-training-intervention design. Over a period of ~15-weeks, participants underwent two 6-week training periods starting with either HIIE or MICE (HIIE–MICE vs. MICE–HIIE). The training programs were of significantly different intensity, but matched for energy expenditure. Each training period consisted of three training sessions per week (on average). Participants underwent a physical fitness assessment before the start of training (week 1), between the two training periods (week 8), and at the end of the study (week 15), including an incremental step test for the standardization of exercise intensity and a VICE session (see Figure 1).

The present manuscript addressed a secondary research question of the iReAct project. As listed in detail above (Hypotheses H1–3), a comparison of acute affective response and potential influencing factors was made among three different endurance exercise modalities, which were linked to the intensity domains described within DMT (MICE ≙ moderate domain, VICE ≙ heavy domain, HIIE ≙ severe domain; Ekkekakis et al., 2005a). Our focus of interest was the *in situ* assessments, which were conducted at four time points (t_0_-t_3_) in three exercise sessions per exercise modality (Ses_1−9_), resulting in a within-subject design with a total number of 36 observations per participant (see Figure 1). Therefore, it is important to note that the group comparison between training type effects across sequences (i.e., MICE–HIIE vs. HIIE–MICE) was not relevant for the current study, and that the following description refers to the relevant aspects for the present analysis. Further details on the clinical trial can be found in the study protocol (Thiel et al., 2020).

### Eligibility and Recruitment

The targeted study group consisted of insufficiently active adults at the time of recruitment following the health-enhancing physical activity recommendations of the World Health Organization (WHO). That is, <150 min/week of moderate physical activity, <60 min/week of leisure-time exercise (including sports participation, endurance-oriented activities, and muscle strengthening), and no regular exercise engagement for several weeks during the last 6 months. Non-adherence to the WHO recommendations was assessed using the validated German version of the European Health Interview Survey—Physical Activity Questionnaire (EHIS-PAQ; Finger et al., 2015). Further inclusion criteria were (a) age from 20 to 40 years, (b) body mass index (BMI) from 18.5 to 30.0 kg/m^2^, (c) non-smokers, (d) maximal oxygen uptake ($\dot{\text{V}}$O_2_max) from 25 to 50 ml/min/kg, (e) no current or former eating disorder or obesity, (f) no severe internistic or neurological previous illness, (g) no pregnancy or breastfeeding period, and (h) German as a native language. Reasons for exclusion from the iReAct study included the following:

- Chronic diseases or findings that result in a decreased ability to exercise
- Medication or supplement intake within the previous 4-weeks
- Counter indication(s) for local anesthetics
- Clinically relevant deviations in the lab results
- Pathological indications in the resting electrocardiogram
- Vein conditions that do not allow for multiple blood sampling
- Participation in a medication study within the last 3 months
- History of drug use or alcohol abuse
- Current psychotherapy.

Recruitment occurred in six consecutive waves over a 2-year period (March 2018 to March 2020). Eligibility was assessed during a telephone screening, as well as a medical examination prior to final enrollment in the study. A total of 58 participants were assessed for eligibility, 49 of whom were included in the randomization process and nine were excluded during medical diagnosis. Out of these nine excluded participants, two were excluded due to time management issues and seven for not meeting the inclusion criteria (gastrointestinal issues [*n* = 2], iron deficiency anemia [*n* = 2], under psychological treatment [*n* = 1], drug consumption [*n* = 1], and BMI above the predetermined upper limit [*n* = 1]). One female, who exhibited a BMI below the specified range, was also included due to her normal percent body fat of 23.5 (normal range: 18–28%) as measured at baseline. The included participants (*N* = 49) were provided with detailed information about the study procedure and associated risks prior to giving written informed consent. During the baseline assessment, five participants dropped out for different reasons (migraine episode [*n* = 1], lung condition being discovered [*n* = 1], time management issues [*n* = 1], lack of willingness to continue participation [*n* = 1], and withdrawal during the VICE session due to discomfort with the exercise [*n* = 1]). Two other participants did not complete the first training period due to illness and thus not being able to complete the minimum adherence. Two non-native speakers included in deviation from study protocol were subsequently excluded from this data analysis because comprehension of the questionnaires could not be guaranteed. Thus, the final study sample comprised 40 insufficiently active healthy adults (men and women) from 20 to 40 years of age. An overview of participants' demographic and anthropometric characteristics can be found in Table 1.

**Table 1:** Demographic and anthropometric characteristics of participants at baseline (*N* = *40*).

**Characteristic**	***M* ±*SD***	**Range**
Age (years)	27 ± 6	20–40
Gender (female/male)	40^a^	29 (72%)/11 (28%)^b^
Height (cm)	171.2 ± 9.1	155.0–190.0
Weight (kg)	69.4 ± 11.1	45.0–101.4
BMI (kg m^−2^)	23.6 ± 2.6	17.6^c^–30.3^d^
$\dot{\text{V}}$O_2max_ (ml kg^−1^ min^−1^)	31.4 ± 4.2	24.2–41.4
HR_max_ (b min^−1^)	191.3 ± 10.8	168.0–207.0
PO_peak_ (W)	162 ± 26	112–217
LTP1 (W)	68 ± 18	35–116
LTP2 (W)	122 ± 22	75–171

*BMI, body mass index; $\dot{\text{V}}$O_2max_, maximal oxygen uptake; HR_max_, maximal heart rate; PO_peak_, peak power output; LTP1, first lactate turning point; LTP2, second lactate turning point*.

a *Total number of participants*.

b *number (percentage) of females/males. Minimum and maximum values of BMI are outside the specified range for one participant each but:*

c *had a normal percentage of body fat (23.5%), and*

d *had a BMI below 30 at inclusion*.

### Sample Size

We did not reach the sample size of *N* = 60 as originally projected in our power calculation and as documented in the study protocol (Thiel et al., 2020). This calculation, however, aimed at group comparisons between training type effects across the two training sequences (i.e., MICE–HIIE vs. HIIE–MICE), which was not the focus of the current study. No separate power analysis was performed for this secondary research question. A *post-hoc* sensitivity analysis (with the given sample size of *N* = 40) suggests that using a simple *t*-test for matched pairs and assuming a type I error of 0.05 (2-sided) and a power of 80% effect sizes of *d* = 0.45 can be detected (no Bonferroni adjustment) (G^*^Power Version 3.1.9.6). We further discuss this issue in the limitations section.

### Measures

#### Affective Response

*Core affective valence*, as the primary outcome variable, was assessed using the validated German version of the Feeling Scale (FS; Hardy and Rejeski, 1989; Maibach et al., 2020). The FS is a single-item, 11-point bipolar rating scale, ranging from −5 (*very bad*) through 0 (*neutral*) to +5 (*very good*) developed for the assessment of affective response during exercise along a displeasure-pleasure continuum.

#### Cognitive Factors

Two cognitive factors were examined in this study. First, *perceived competence* (PC) was operationalized via the level of agreement regarding the statement “*I feel like I am very competent for the physical activity.”* This single item has been formulated in context-specific variation by Sudeck and Conzelmann (2014). The version used in the present study was based on a 7-point bipolar rating scale ranging from 1 (*strongly disagree*) to 7 (*strongly agree*). Second, *awareness of interoceptive cues* (AOI) was assessed using a single-item designed on the basis of Rose and Parfitt's (2010) procedures. Participants were asked to rate the influence of interoceptive cues on their general affective state during exercise by completing the statement “*My physical reactions and sensations were*...” on a visual analog scale ranging from 0 to 100 with three verbal anchor points: *very disturbing* (0), *neutral* (50), and *very beneficial* (100).

#### Interoceptive Factor

The extent of interoceptive stimuli was operationalized by *heart rate* (HR), which was constantly monitored through a HR belt (further information follows in the next section). Minor artifacts in the HR data were cleaned using an anomaly detection algorithm to delete noisy data points (implausible spikes or gaps due to technical problems), which uses the interquartile method to find outliers (Upton and Cook, 1996). Each noisy data point was deleted, and the HR was then interpolated on a second-by-second basis.

### Procedures

#### Standardization of Exercise Intensity

In the laboratory visits (weeks 1, 8, and 15; see Figure 1), participants undertook an incremental step test to volitional exhaustion on a cycle ergometer (Ergoselect 200; Ergoline GmbH, Bitz, Germany) for determination of the $\dot{\text{V}}$O_2max_, peak power output (PO_peak_), and lactate thresholds (first lactate turning point [LTP1] and second lactate turning point [LTP2]). Before starting the test, baseline blood pressure and capillary blood lactate concentration ([La^−^]) were measured. The test began with a 2-min resting period on the bike, followed by 25-watt (W) step increments every 3 min, starting at 50 W for males and at 25 W for females, until task failure. [La^−^] was analyzed (Biosen S-Line; EKF, Cardiff, UK) by collecting capillary blood samples (20 μL) from the right earlobe during the last 20 s of each stage and immediately after volitional exhaustion. HR and electrocardiogram (ECG) were constantly monitored throughout the test (12-channel PC ECG; custo med GmbH, Ottobrunn, Germany). Breath-by-breath pulmonary gas exchange and ventilation ($\dot{\text{V}}$E) were measured using a metabolic cart (MetaLyzer; CORTEX Biophysics, Leipzig, Germany). Calibration was performed before each test following the manufacturer's instructions. Lactate thresholds were analyzed using a segmented regression model at which two breakpoints were estimated from the [La^−^]–power output relationship. LTP1 was determined as the first rise in [La^−^] above baseline levels (first breakpoint), which is accompanied by the first increase in $\dot{\text{V}}$E as a function of $\dot{\text{V}}$O_2_ (i.e., VT1). LTP2 was determined as the second abrupt increase in [La^−^] (second breakpoint), which is accompanied by the second sharp increase in $\dot{\text{V}}$E as a function of $\dot{\text{V}}$O_2_ (i.e., VT2; Binder et al., 2008; Hofmann and Tschakert, 2017).

#### Exercise Modalities

**Figure 2:**
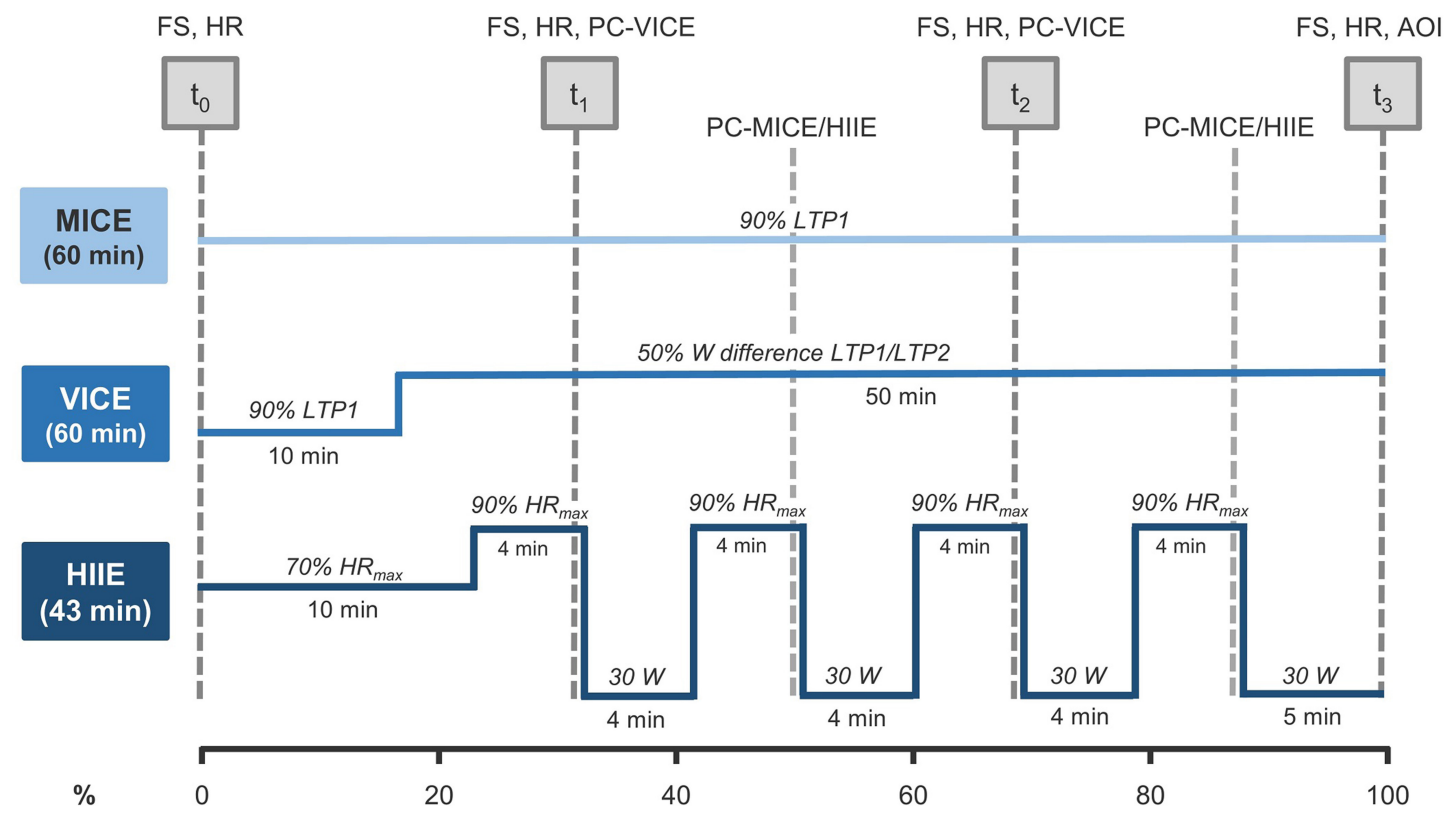
Overview of exercise modalities and measures. Data was collected at 4 points in time: pre-exercise (t_0_), in-task (t_1_, t_2_), and post-exercise (t_3_). The x-axis describes the percentage of exercise completed (0–100%). FS, Feeling Scale; HR, heart rate; PC-VICE, perceived competence assessed in VICE; PC-MICE/HIIE, perceived competence assessed in MICE/HIIE; AOI, awareness of interoceptive cues; MICE, moderate-intensity continuous exercise; VICE, vigorous-intensity continuous exercise; HIIE, high-intensity interval exercise; LTP1, first lactate turning point; LTP2, second lactate turning point.

All exercise modalities were performed on calibrated bicycle ergometers (ec5000; custo med GmbH, Ottobrunn, Germany). Based on the results of spiroergometric and lactate measurements, the following exercise protocols were defined (for a graphical illustration, see Figure 2):

(a) *MICE* was prescribed as 60 min of continuous cycling at the power output (PO) corresponding to 90% of LTP1 (≙ moderate-intensity domain).
(b) *VICE* was performed for 50 min at a constant PO corresponding to the midpoint between the first and the second lactate threshold (i.e., 50% of the difference between LTP1 and LTP2; ≙ heavy-intensity domain). The session was introduced by a 10-min warm-up at a PO corresponding to 90% of LTP1 (≙ intensity of MICE), totaling 60 min of exercise.
(c) *HIIE* involved 4 x 4-min intervals at a PO corresponding to 90% of HR_max_. This exercise intensity was chosen as such intensity would be within the severe-intensity domain for this population (i.e., all the exercise intensities were above LTP2). Each high-intensity load interval was interspersed with a 4-min active recovery at 30 W. The session was enclosed by a 10-min warm-up (at 70% of HR_max_) and a 5-min cool-down at 30 W, totaling 43 min of exercise duration.

All exercise sessions were supervised by trained personnel. While VICE took place under standardized controlled laboratory conditions, MICE and HIIE were completed in a health and fitness orientated training environment. However, we considered a potential modality-dependent influence of environment-related characteristics on the affective response in preliminary analyses (see Supplementary Table 1).

### Data Collection

An overview of measures used at the different survey time points can be seen in Figure 2. Within each survey session, affective valence (measured by FS) was recorded prior (t_0_), at two time points in-task (t_1_, t_2_), and immediately after exercise (t_3_) (i.e., four time points in total). In-task assessments for FS were performed after 14 and 30 min in HIIE (i.e., in the last 15 s of the first and third loading intervals). To achieve temporal alignment of the measurement time points, minutes 20 and 40 were set for the continuous exercise modalities (MICE and VICE). Cognitive factors were assessed during and after the exercise session. Data collection points for PC were minutes 22 and 38 in HIIE (i.e., in the last 15 s of the second and forth loading interval), minutes 20 and 40 in VICE, and minutes 31 and 53 in MICE. The time-lagged assessment in MICE and HIIE resulted from other study interests as well as the rationale of not overloading individual measurement time points. Consequently, PC values in MICE/HIIE were estimated using the next observation carried backward (NOCB) method (Engels and Diehr, 2003). AOI was collected in the form of a retrospective rating immediately after exercise cessation (see Figure 2).

In-task assessments were implemented by presenting the individual items on A3 posters as visual references so that the participants could concentrate on the exercise itself. The questions, including scale anchors, were read aloud by the investigator and the participant's response was recorded via smartphone (Google Nexus 5; LG Group, Seoul, South Korea) with the movisensXS application (movisens GmbH, Karlsruhe, Germany) after consultation. Pre- and post-surveys were conducted independently by the participants with smartphone in hand. HR was collected throughout all sessions using a HR belt (3-channel ECG; custo med GmbH, Ottobrunn, Germany). Based on this training monitoring, HR was adjusted to fitness changes over the weeks (for details, see Mattioni Maturana et al., 2021b). This ensured that participants were always exercising within the originally prescribed relative intensity of exercise.

### Statistical Analyses

A manipulation check was carried out to verify whether the participants were exercising at different exercise intensities in the three exercise modalities as intended. Separate one-way analysis of variance (ANOVAs) were executed for the variables of %HR_max_ and %HRR.

Descriptive statistics using means (*M*) and standard deviations (*SD*) were generated for continuous variables according to the distribution; frequencies (*n*) and percentages (%) were generated for categorical variables. Intraclass correlation coefficients (ICCs) were calculated for the main outcome measures. Furthermore, estimates of the within-person variability across the FS measurements (9 sessions × 4 time points) and the PC, AOI, and HR measurements (9 sessions × 3 time points), as well as the between-person variability in these outcomes, were calculated.

For the first research question regarding in-task (t_1_, t_2_) affective valence in the three different exercise modalities (Hypothesis H1a), we fit a multilevel model for repeated measures with the levels *subject* (ID), *session* (s1, s2, s3), and the crossed factor *modality* (MICE, VICE, HIIE) to examine the effect of intensity conditions during the intervention on the FS (Model 1). A step-up model construction strategy was applied, retaining fixed effects in the model if they demonstrated statistical significance (*p* < 0.05) and successively adding random effects to account for the correlated structure of the data. Due to non-convergence caused by over-parameterization (the random effects structure exhibited a complexity not supported by the underlying data), we did not account for the nesting of individuals within group sequence (i.e., MICE-HIIE vs. HIIE-MICE). Thus, our final model included the fixed effect modality and a random intercept on the subject and session level, as well as allowing for a random slope for exercise modalities. To examine the in-task (t_1_, t_2_) variability of affective valence in the three different exercise modalities (Hypothesis H1b), equality of variances between exercise modalities was tested using the median-based Levene's test.

To address the second research question with regard to the course of the affective valence across a session within the three different exercise modalities (Hypothesis H2), data from the pre-exercise time point (t_0_), the in-task time points (t_1_, t_2_), and the post-exercise time point (t_3_) were examined in the model (Model 2). As fixed effects, we considered modality, time point, and the interaction term modality *x* time point. A random intercept on session and subject level, as well as a random slope, were included.

For the third research question concerning the identification of exercise modality-dependent predictors of in-task (t_1_, t_2_) affective response (Hypothesis H3), we extended Model 1 by separately introducing one of three factors (PC, AOI, or HR) and its interaction with modality as fixed effects (Model 3a, b, c, respectively).

Due to the low numbers of units on the subject level, simple covariance structures (scaled identity) had to be chosen to reach convergence in all the models. Significant effects were followed by pairwise *post hoc* comparisons applying Bonferroni adjustments. For significant interaction terms, *post hoc* probing was performed to describe the direction of the interaction effect. We contrasted effects with one *SD* below and above the mean value (± 1 *SD*) using two-way interaction plots.

Data preparation and statistical analyses were carried out using the Statistical Package for Social Sciences (SPSS, version 26; IBM Corp., Armonk, NY, USA). All *p*-values were two-sided, and the statistical significance level was set at *p* < 0.05.

## Results

### Descriptive Analyses

The manipulation check confirmed that participants were exercising at different intensities in the three exercise modalities based on mean in-task %HR_max_, *F*_(2,645)_ = 701.53, *p* < 0.001, η^2^ = 0.69, and %HRR, *F*_(2,645)_ = 643.92, *p* < 0.001, η^2^ = 0.67. In addition, comparison of the HR data with reference values proposed by Binder et al. (2008) indicated that the exercise modalities were within the targeted intensity domains (for descriptive statistics see Supplementary Table 2).

Descriptive statistics of the study variables can be found in Table 2. It is important to note that there are missing values due to disturbances in HR measurement as well as an early termination of the last survey wave due to the COVID-19 pandemic. The grand means of FS, PC, and AOI were in the upper third or upper half of the respective scales. The empirical range of person means varied from −0.12 to 4.46 for FS, from 2.70 to 6.89 for PC, and from 33.11 to 85.11 for AOI, indicating substantial between-person variability. The ICCs indicated that 35% (FS), 63% (PC), or 47% (AOI) referred to between-person differences. In contrast, the ICC of HR indicated that 75% could be attributed to within-person differences.

**Table 2:** Descriptive statistics for study variables.

**Variable**		**Between-person variability**				**Within-person variability**			
	**ICC**	***N***	***M***	***SD***	**Range**	***N***	**mV**	***SD***	**Range**
FS [−5 to +5]	0.35	40	2.70	0.96	−0.12 to 4.46	1360^a^	80	1.29	0.17 to 2.66
PC [1 to 7]	0.63	40	5.29	1.13	2.70 to 6.89	1020^b^	60	0.85	0.19 to 2.37
AOI [0 to 100]	0.47	40	56.10	12.83	33.11 to 85.11	1020^b^	60	13.14	3.03 to 27.41
HR	0.25	40	149.74	12.01	122.35 to 169.34	958^b^	122	19.48	13.03 to 26.49

*ICC, intraclass correlation coefficient; mV, missing values; FS, Feeling Scale; PC, perceived competence; AOI, awareness of interoceptive cues; HR, heart rate*.

a *FS was measured at 4 points in time (pre-exercise [t_0_], in-task [t_1_, t_2_], and post-exercise [t_3_])*.

b *PC, AOI, and HR were measured at 3 points in time (t_1_, t_2_, and t_3_)*.

### Main Analyses

#### Valence and Variation of In-task Affective Response

**Figure 3:**
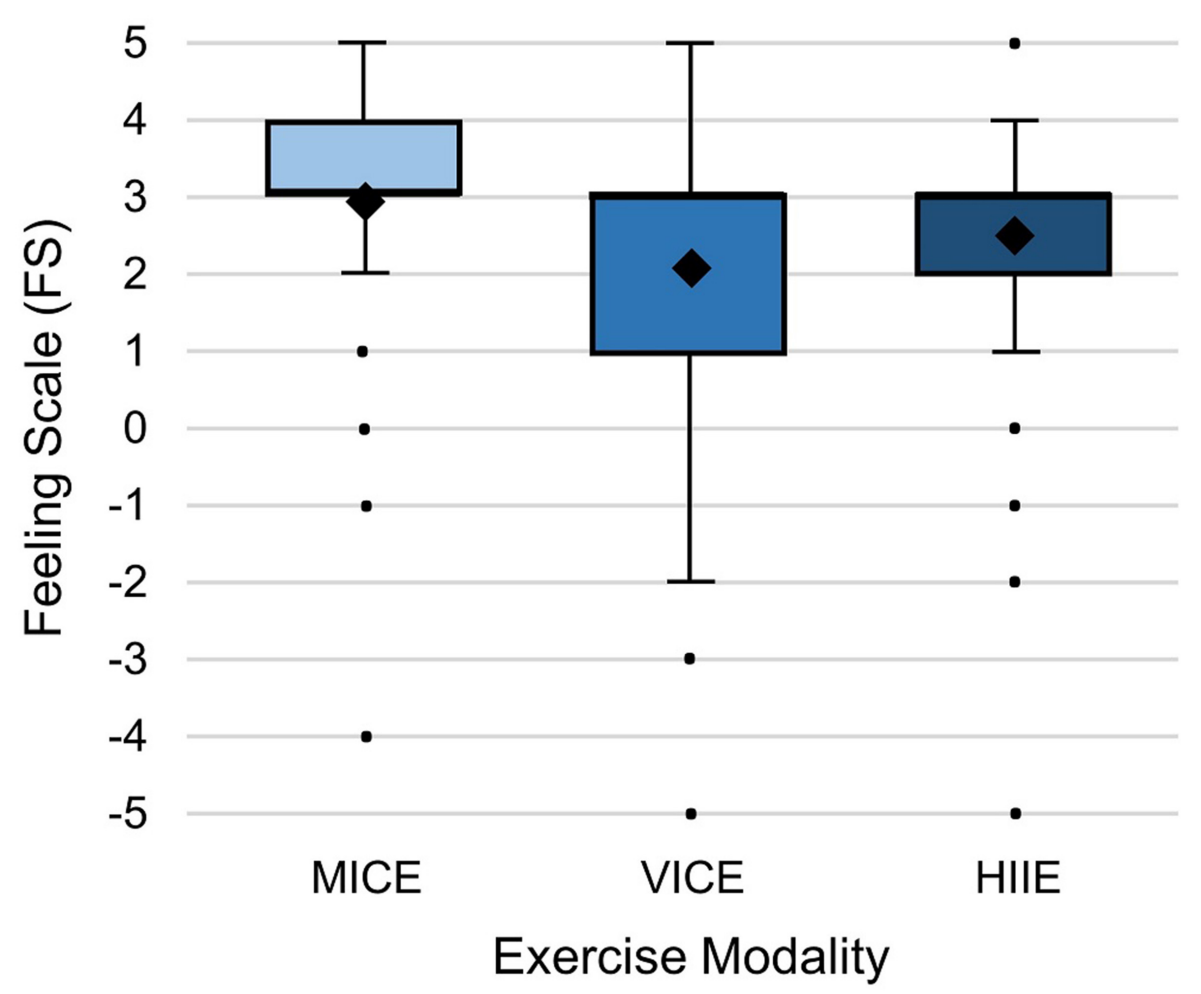
Affective valence (Feeling Scale) and its variation within the three different exercise modalities: MICE, moderate-intensity continuous exercise; VICE, vigorous-intensity continuous exercise; HIIE, high-intensity interval exercise. The diamonds represent the mean values. Raw data is presented for the in-task time points (t_1_, t_2_) of each of the three sessions (Ses_1−3_) without adjustment for the dependencies within clusters.

Figure 3 illustrates the differences of in-task (t_1_, t_2_) affective valence and its variation among the three different exercise modalities (the corresponding descriptive statistics are provided in Supplementary Table 3). Model 1 revealed a significant main effect for modality (*F* = 14.56, *p* < 0.001) on affective valence (FS). Pairwise *post hoc* comparisons (see Table 3) showed FS to be significantly higher in MICE than in VICE and HIIE. No significant difference was found between VICE and HIIE (confirmation of Hypothesis H1a). Levene's test results indicated significant variance differences among exercise modalities (*F* = 8.57, *p* < 0.001). Pairwise *post hoc* comparisons showed significantly higher variability of FS in VICE than in MICE (*F* = 19.91, *p* < 0.001; confirmation of Hypothesis H1b) and in HIIE (*F* = 8.98, *p* = 0.003). No differences in variability were found between MICE and HIIE (*F* = 2.52, *p* = 0.113).

**Table 3:** Associations of exercise modality and psychophysiological factors with affective valence.

		**Fixed effect**	**B**	**SE**	***p***
**MODEL 1: in-task affective valence** ^**a**^					
		MICE vs. VICE	0.886	0.165	<0.001^***^
	Exercise Modality	MICE vs. HIIE	0.535	0.166	0.005^**^
		VICE vs. HIIE	−0.350	0.166	0.114
**MODEL 3: Predictors of in-task affective valence** ^**b**^					
	PC × Exercise Modality	VICE vs. MICE	0.342	0.107	0.006^**^
(a)		MICE vs. HIIE	−0.051	0.108	>0.999
		VICE vs. HIIE	0.291	0.088	0.003^**^
	AOI × Exercise Modality	VICE vs. MICE	0.013	0.007	0.186
(b)		MICE vs. HIIE	0.006	0.007	>0.999
		VICE vs. HIIE	0.019	0.007	0.015^*^
	HR × Exercise Modality	VICE vs. MICE	−0.023	0.009	0.033^*^
(c)		MICE vs. HIIE	−0.009	0.011	>0.999
		VICE vs. HIIE	−0.032	0.010	0.003^**^

*The results represent pairwise post hoc comparisons of Feeling Scale (FS) values. PC, perceived competence; AOI, awareness of interoceptive cues; HR, heart rate; x, interaction term; MICE, moderate-intensity continuous exercise; VICE, vigorous-intensity continuous exercise; HIIE, high-intensity interval exercise*.

a *In Model 1, we examined in-task (t_1_, t_2_) affective valence by including the levels subject, exercise session, and the crossed factor exercise modality (MICE, VICE, HIIE)*.

b *For Model 3, we extended Model 1 by separately introducing the interaction term of one of three potential predictors (3a: PC, 3b: AOI, 3c: HR) with exercise modality (x exercise modality) as a fixed factor*.

* *p < 0.05*.

** *p < 0.01*.

*** *p < 0.001 (Bonferroni adjusted)*.

#### Course of Affective Response Across the Session

**Figure 4:**
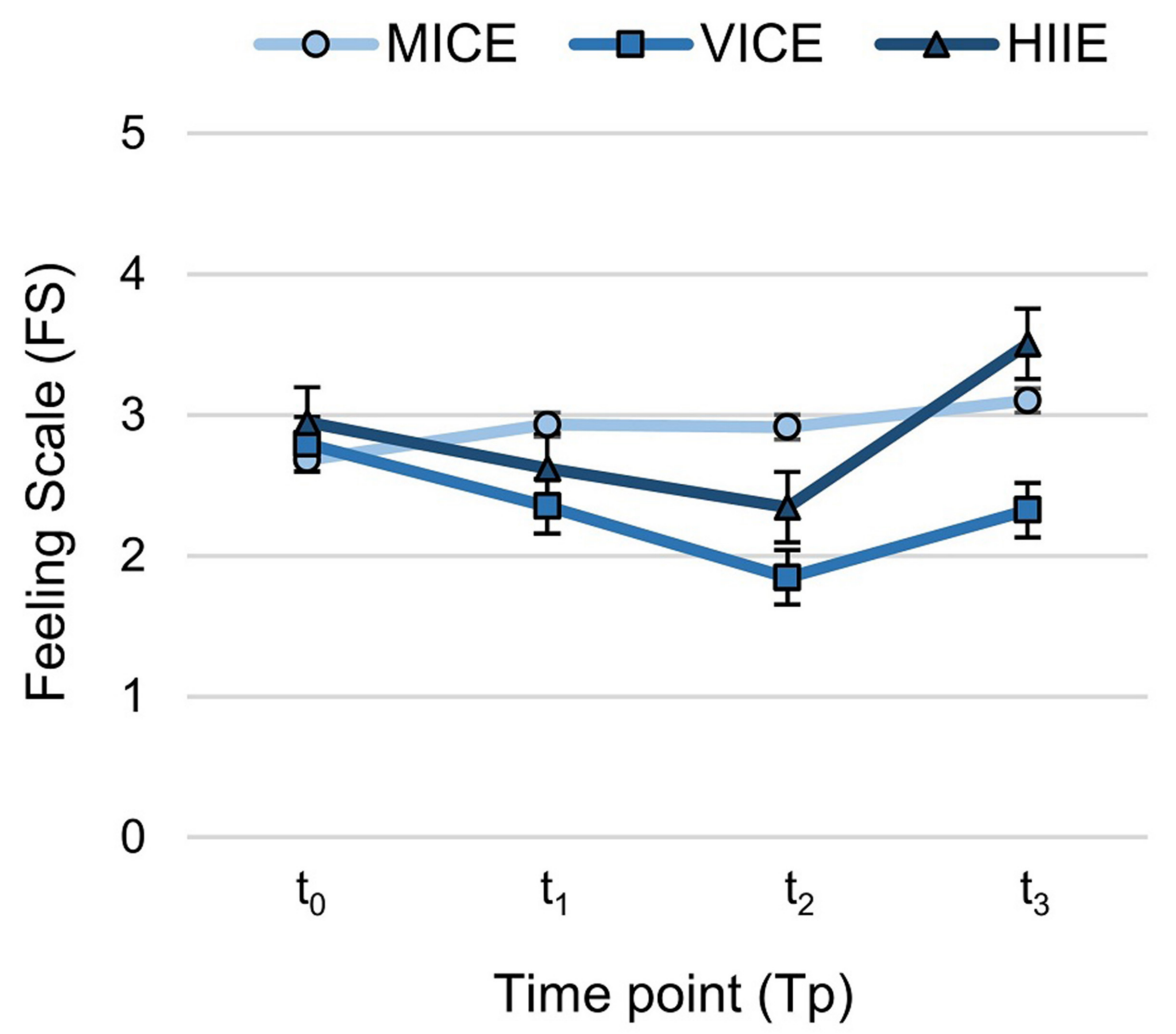
Course of affective valence (Feeling Scale) across the session within the three different exercise modalities: MICE, moderate-intensity continuous exercise; VICE, vigorous-intensity continuous exercise; HIIE, high-intensity interval exercise. The graphs represent estimated means and standard errors (Model 2) over 4 time points (pre-exercise [t_0_], in-task [t_1_, t_2_], and post-exercise [t_3_]).

Figure 4 shows that, while a slight increase of FS occurred in MICE, there was an affective rebound in VICE and HIIE. Model 2 revealed significant main effects for modality (*F* = 6.08, *p* = 0.003) and time point (*F* = 24.22, *p* < 0.001) on FS; however, they were qualified by the interaction modality *x* time point (*F* = 13.33, *p* < 0.001). In the pairwise *post hoc* analysis (see Table 4), we observed a significant increase of FS from pre- to post-exercise in MICE. In contrast, a significant decrease in FS from pre-exercise to t_1_ and t_2_ was evident for VICE and HIIE, followed by a significant increase from t_2_ to post-exercise (confirmation of Hypothesis H2). While the FS post value for VICE remained below the pre-exercise value, the FS post value for HIIE increased above baseline.

**Table 4:** Comparisons of affective valence at four time points within exercise modalities.

	**Tp**	**Δ_M_**	**SE**	***p***
MICE	t_1_ vs. t_0_	0.250	0.107	0.119
	t_2_ vs. t_0_	0.233	0.126	0.390
	t_3_ vs. t_0_	0.422	0.144	0.021^**^
	t_2_ vs. t_1_	−0.017	0.090	>0.999
	t_3_ vs. t_1_	0.172	0.114	0.791
	t_3_ vs. t_2_	0.190	0.132	0.913
VICE	t_1_ vs. t_0_	−0.441	0.109	<0.001^***^
	t_2_ vs. t_0_	−0.946	0.129	<0.001^***^
	t_3_ vs. t_0_	−0.468	0.147	0.009^***^
	t_2_ vs. t_1_	−0.505	0.092	<0.001^***^
	t_3_ vs. t_1_	−0.027	0.117	>0.999
	t_3_ vs. t_2_	0.477	0.135	0.003^***^
HIIE	t_1_ vs. t_0_	−0.327	0.108	0.016^**^
	t_2_ vs. t_0_	−0.602	0.128	<0.001^***^
	t_3_ vs. t_0_	0.558	0.146	0.001^***^
	t_2_ vs. t_1_	−0.274	0.092	0.018^**^
	t_3_ vs. t_1_	0.885	0.116	<0.001^***^
	t_3_ vs. t_2_	1.159	0.134	<0.001^***^

*The results represent pairwise post-hoc comparisons of Feeling Scale (FS) values for the 4 points in time (Tp: pre-exercise [t_0_], in-task [t_1_, t_2_], and post-exercise [t_3_]; Model 2). MICE, moderate-intensity continuous exercise; VICE, vigorous-intensity continuous exercise; HIIE, high-intensity interval exercise*.

* *p < 0.05*.

** *p < 0.01*.

*** *p < 0.001 (Bonferroni adjusted)*.

#### Psychophysiological Predictors of In-task Affective Response

**Figure 5:**
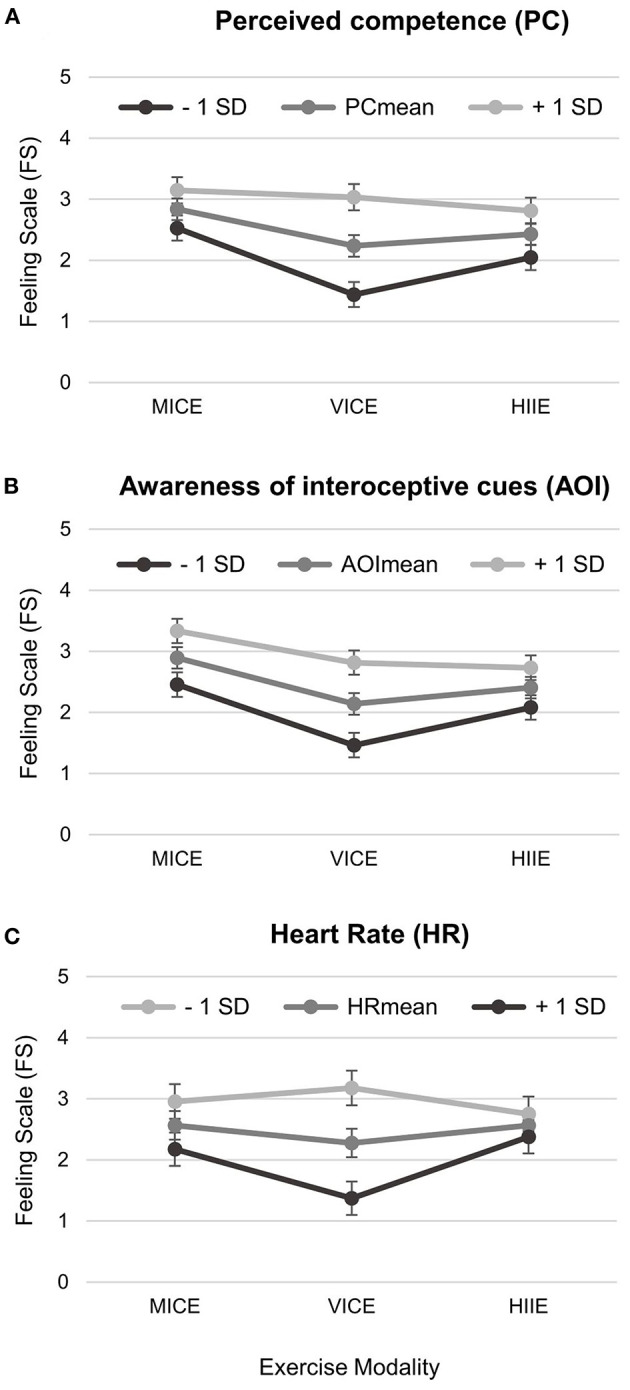
Two-way interaction plots of psychophysiological predictors of in-task (t_1_, t_2_) affective valence (Models 3a-c): **(A)** Perceived competence, **(B)** Awareness of interoceptive cues, and **(C)** Heart rate. Shown is the respective expression of the Feeling Scale (FS) for the mean value of the predictor as well as for one standard deviation below the mean value (−1 SD) and for one standard deviation above the mean value (+1 SD) with error bars representing standard errors. MICE, moderate-intensity continuous exercise; VICE, vigorous-intensity continuous exercise; HIIE, high-intensity interval exercise.

Figure 5 depicts the association of cognitive (PC, AOI) and interoceptive (HR) factors with FS as a function of exercise modality. Models 3a-c revealed significant main effects for all fixed factors and interaction terms.

For PC (Model 3a), we found significant main effects for modality (*F* = 10.58, *p* < 0.001) and PC (*F* = 41.54, *p* < 0.001) on FS; however, they were qualified by the interaction modality *x* PC (*F* = 7.55, *p* = 0.001). Pairwise *post hoc* comparisons (see Table 3) showed a significantly stronger association of PC with FS in VICE than in MICE (confirmation of Hypothesis H3) and in HIIE, while no significant difference was observed between MICE and HIIE. Contrasting the interaction effect with one *SD* higher and lower in PC resulted in an increase and decrease, respectively, on the FS by 0.80 in VICE vs. 0.31 in MICE and 0.38 in HIIE (see Figure 5A).

For AOI (Model 3b), we observed significant main effects for modality (*F* = 8.40, *p* = 0.001) and AOI (*F* = 58.29, *p* < 0.001) on FS; however, they were qualified by the interaction modality *x* AOI (*F* = 4.29, *p* = 0.014). Pairwise *post hoc* comparisons (see Table 3) revealed a significantly stronger association of AOI with FS in VICE than in HIIE. In contrast, no significant differences were found between VICE and MICE (rejection of Hypothesis H3) or between MICE and HIIE. Effect contrasting of AOI resulted in an FS change of 0.66 in VICE vs. 0.32 in HIIE (and 0.43 in MICE; see Figure 5B).

For HR (Model 3c), the model revealed significant main effects for modality (*F* = 5.33, *p* = 0.006) and HR (*F* = 17.61, *p* < 0.001) on FS; however, they were qualified by the interaction modality *x* HR (*F* = 6.61, *p* = 0.002). Pairwise *post hoc* comparisons (see Table 3) showed a significantly stronger association of HR with FS in VICE than in the two other modalities (MICE and HIIE), where no significant difference was observed. Effect contrasting of HR resulted in a FS change of 0.90 in VICE vs. 0.39 in MICE and 0.19 in HIIE (see Figure 5C). Importantly, the direction of the interaction effect here was opposite to that for the cognitive factors, such that an increase in HR rate was accompanied by a decrease in affective valence.

## Discussion

The present study was designed to investigate acute affective response associated with endurance exercise modalities considering different exercise intensities. Based on the lactate threshold concept proposed by DMT, two continuous exercise protocols within the moderate- (MICE) or heavy-intensity domain (VICE) and an interval exercise in the severe-intensity domain (HIIE) were compared in a within-subject study among insufficiently active adults. Basically, the current findings provide support for the tenets of DMT regarding continuous exercise, but suggest that these are not directly applicable to the intermittent nature of HIIE that allows periods of recovery between bouts of severe exercise.

Consistent with the hypotheses, in-task affective valence was more positive in MICE compared with VICE and HIIE, while there was no significant difference between the latter two. However, the descriptive statistics suggested that VICE was more negatively valenced in comparison with HIIE. Taken together, our results are in line with previous research, indicating similar (Martinez et al., 2015; Niven et al., 2018; Alicea et al., 2020) or even more positive affective responses (Jung et al., 2014; Kilpatrick et al., 2015; Martinez et al., 2015) in HIIE as opposed to VICE. On the basis of these results, it can be assumed that the short periods of severe intensity in HIIE are not of sufficient duration to disrupt homeostasis to such an extent that a more negative affective response is induced. Rather, the rest periods seem to mitigate the detrimental effect associated with the HIIE load intervals. Reduced monotony, the prospect of getting a break, and a feeling of pride after the completion of each interval could positively influence the affective response in contrast to continuous exercise in the heavy domain. In line with this assumption, studies showed that the specific characteristics of HIIE promote participants' self-efficacy beliefs (Jung et al., 2014) and result in more positively valenced post-exercise narrative responses (e.g., feelings of reward and reenergization associated with the rest interval) in comparison with continuous exercise (Alicea et al., 2020). Due to the fact that, in our study, we exclusively considered affective response at the very end of the load intervals, it seems plausible that the beneficial influence of the interval exercise modality in terms of affective valenced states was not only reflected in the overall picture but also within the specific severe-intensity exercise periods.

Further, a rebound to more positive affect was observed in this study following exercise in the heavy and severe domain, which aligns with the assumptions of DMT (Ekkekakis et al., 2005a) and empirical evidence (e.g., Stork et al., 2018; Alicea et al., 2020; Box et al., 2020). As hypothesized for the course across the session, MICE was associated with a slight increase in affective valence from pre- to post-exercise, whereas VICE and HIIE caused a decline in pleasure, followed by an affective rebound immediately after exercise termination. Regarding the amount of affective rebound, post values of affective valence in our study remained below baseline values in VICE, but exceeded baseline values in HIIE. Importantly, the post-value was collected in VICE directly following the exercise, whereas in HIIE, a 5-min cool-down was performed before the end of exercise. Results of previous studies suggested that the postulated affective rebound effect only develops (completely) in the post-exercise period (e.g., Decker and Ekkekakis, 2017; Stork et al., 2018; Box et al., 2020). Thus, it can be assumed that a cool-down phase or a later survey time point of post-exercise affective response (e.g., 5 min post-exercise) would also have revealed a rebound to more positive valenced states in VICE.

The current results revealed the heavy-intensity range (i.e., VICE) as the zone of the highest response variability. Furthermore, the study provided an explanation for this finding by showing a higher importance of psychophysiological factors within the heavy domain. Both cognitive factors (perceived competence and awareness of interoceptive cues) almost consistently had a greater association with affective response during VICE in contrast to MICE and HIIE (see Figures 5A,B). A one *SD* higher score on either cognitive factor resulted in twice the rate of change in affective valence in VICE compared with HIIE. Moreover, perceived competence was shown to be more relevant within continuous exercise in the heavy domain (i.e., VICE) compared with the moderate domain (i.e., MICE), associated with an even 2.5 times higher rate of change in affective valence. Such a tendency could also be seen for the awareness of interoceptive cues with a factor of 1.5 for the comparison of impact in VICE vs. MICE, although here the significance level was not achieved.

There was no difference in the association of cognitive factors on affective response in the moderate (i.e., MICE) and severe (i.e., HIIE) modalities considered here. While previous research based on a comparison of imposed and self-selected HIIE concluded that (reflecting the assumptions of DMT on continuous exercise) affective valence within the severe domain is mediated by exercise intensity rather than the feeling of autonomy (Kellogg et al., 2019), the present results suggest an influence of cognitive factors in HIIE. Thus, it is possible that the intermittent nature of HIIE prevents a switch to a mode of affect induction that relies primarily on interoceptive stimuli. Supporting this assumption, we found an analogous pattern for the modality-dependent association of the interoceptive factor with affective response to that of the cognitive factors studied (see Figure 5C). That is, heart rate had a greater importance in the heavy vs. the other two domains, with a two times (VICE vs. HIIE) or even five times (VICE vs. MICE) higher rate of negative change in affective valence. This finding does not support the proposition of DMT for continuous exercise, that interoceptive stimuli have the greatest (negative) influence in the severe domain. Importantly, the heart rate examined in this study represents only one physiological factor and may have a different influence on affective response in contrast to a neurophysiological (e.g., heart rate variability), cardiorespiratory (e.g., oxygen uptake), or metabolic marker (e.g., blood lactate). For example, a study by Roloff et al. (2020) demonstrated that valenced affective states closely track changes in oxygen uptake in four different HIIE protocols.

Taking a closer look at HIIE, the contradictory study results can potentially be explained by examining the exercise protocol variables, such as work-to-rest ratio, total session duration, and energy expenditure. Since these characteristics decisively determine the extent of reliance on limited energetic resources of the anaerobic metabolism, they are supposed to have a decisive influence on affective response to exercise (Ekkekakis et al., 2005a). Therefore, it is not surprising that the strenuous HIIE protocol in a study from Oliveira et al. (2013) with 2-min work intervals and <1-min recovery periods resulted in less pleasure compared with VICE. Likewise, the detrimental influence of HIIE observed in the study of Decker and Ekkekakis (2017) could have been due to their demanding protocol with a work-to-rest ratio of 1:0.66 and a comparatively high intensity within the recovery periods (85% of VT1). In contrast, the 1:1 work-to-rest ratio used in this and in other studies (e.g., Jung et al., 2014; Alicea et al., 2020), with comparatively lower dependence on anaerobic metabolism, resulted in equal or even more positive affective states in HIIE vs. VICE. Interestingly, the negative impact of longer interval duration of 120 s in HIIE on affective response in comparison with VICE that was found in the study by Martinez et al. (2015) was not evident for this study which had an interval duration of 240 s. However, due to an equation of energy expenditure in their study, HIIE was of longer duration than VICE (24 vs. 20 min), whereas in our study, we used a significantly shorter HIIE protocol compared with VICE (43 vs. 60 min), since the main argument for promoting HIIE in public health is its time efficiency. Nonetheless, there is increasing evidence that low-volume HIIE may diminish feelings of displeasure during exercise (e.g., Haines et al., 2020). Similarly, reducing the intensity of load intervals (from 100 to 85% of peak power) has been shown to be a successful strategy for obtaining more positive affective experiences in HIIE while maintaining a health-promoting heart rate stimulus (Malik et al., 2019).

### Strengths and Limitations

Some strengths of the current study are noteworthy. First, responding to the call for psychophysiological perspectives in research examining the affective response to exercise (Acevedo and Ekkekakis, 2006), we standardized exercise intensities relative to metabolic landmarks with reference to a physiological framework. This allowed us to ensure accurate comparisons of affective response across the three different exercise intensity domains proposed by DMT. Second, in line with current recommendations, we assessed the valence component of basic affect (using FS) before, during, and after the exercise session to provide in-task as well as course-specific patterns of affective response. Third, we applied a mixed model approach to account for the nested data structure of the within-subject design (9 sessions with 4 time points per participant). Finally, by recruiting adults who did not achieve the recommendations for health-promoting physical activity, the current findings are of direct relevance to a segment of the population that is particularly in need of interventions for promoting exercise.

Although the current study produced novel and important findings, some potential limitations should be mentioned. As this study addressed a secondary research question of the iReAct project, no group comparison between training type effects across sequences (i.e., MICE–HIIE vs. HIIE–MICE) was made, thus neglecting potential carry over effects. In addition, it is important to consider that, because the participants completed a 15-week training program, strictly speaking, they were no longer physically inactive during the study. However, through training monitoring, we accounted for fitness changes over the weeks and ensured that participants were exercising within the originally prescribed relative intensities.

Other limitations relate to the *in situ* assessments. First, due to concerns about overloading the surveys, not all measures were collected at each survey time point during exercise, so the PC scores had to be estimated using the NOCB method. Although we consider this missing data approach to be reasonable due to the assumption of a certain latency period of the competence experience, the shift in data may have been associated with an over- or underestimation of effects. Second, for HIIE, we considered only responses within the load intervals (as representants of the severe-intensity domain). However, looking at the whole, the intermittent nature of interval exercise seems to play an important role (Stork et al., 2018). Especially when it comes to predicting subsequent exercise behavior on the basis of in-task response, fluctuations in affect during both the rest and load intervals should be considered in future studies. Third, the 5-min cool-down period in HIIE compared with VICE made it difficult to compare the extent of the affective rebound. It would certainly have been worthwhile to have considered affective valence not only immediately after the end of exercise, but also at several time points in the post-exercise period (e.g., 10, 20, and 30 min post-exercise; Decker and Ekkekakis, 2017), but this was not possible due to other study interests. Last, *in situ* assessments were carried out in different environmental conditions with a rather sterile laboratory setting (VICE) on the one hand and a less standardized training area (MICE and HIIE) on the other. Although we took this potential confounder into account in preliminary analyses, a minor influence of environment-related characteristics on affective response to exercise cannot be completely ruled out.

Moreover, the three exercise modalities assessed in the present study should be considered as only a selection of a large pool of possible options. In particular, we solely examined one HIIE protocol, so no conclusions can be drawn about which specific variables of HIIE influenced acute affective response in our study. Because there is high variability in HIIE protocol configurations among studies that limits the generalizability of our and previous findings, future research should emphasize a more detailed comparison of different HIIE sessions to determine optimally configured protocols. A balanced work-to-rest ratio as well as shorter total duration compared with continuous exercise seems to be promising.

Lastly, only 40 participants could be recruited for the elaborate within-subject design with 15-weeks of training and three extensive diagnostic blocks that did not allow for extended absences. Due to this low sample size, observed effects have to be interpreted with caution. In future studies, accounting for interindividual differences, it should be tested how stable these effects are, in order to improve the generalizability of the results. In this context, due to the highly standardized ergometer training, the limited external validity should be mentioned as another limiting factor.

### Practical Implications and Conclusion

The current findings provide us with a more comprehensive understanding of insufficiently active people's acute affective response to MICE, VICE, and HIIE. When it comes to aerobic exercise prescriptions, one size does not fit all due to interindividual differences. Thus, health promotion practitioners should offer beginners the opportunity to try different forms of endurance exercise in order to achieve more positive affective states. In addition to this acute experimental manipulation study of affective response to prescribed endurance exercise modalities, future studies should expand the scope of the present investigation to real-world settings (i.e., increase external validity) and long-term exercise adherence monitoring.

In the process of personalized exercise programming, trait differences (i.e., individuals' preference for and tolerance of exercise intensity or modality; Ekkekakis et al., 2005b) as well as state differences (i.e., individuals' pre-exercise physical and mental “readiness-to-exercise”; Strohacker and Zakrajsek, 2016) need to be considered. Thus, HIIE may be a viable, time-efficient strategy for some individuals and certain occasions in obtaining positive psychological responses and long-term health benefits. A combination of both HIIE and continuous exercise should be considered, in order to better utilize the advantages of each modality and to bring more variety and flexibility into the daily exercise routine.

Our results suggest that, in addition to bolstering one's self-perception of competence, changing individual interpretations of interoceptive cues (i.e., cognitive reframing) may be one avenue to increase pleasure during exercise, especially in the heavy intensity domain. Consequently, in addition to dissociative strategies of directing attention away from bodily symptoms (i.e., producing an external attentional focus by exercising with music/video; Karageorghis et al., 2021 or exercising in natural environments; Bourke et al., 2020), there is also potential for the use of associative strategies that consciously address the (unpleasant) bodily sensations themselves (e.g., mindfulness practices during exercise; Cox et al., 2020).

Collectively, this study provides insight into how exercise can be structured to elicit more positive affective states, and contributes to a theory-based foundation for the development and implementation of more individualized exercise promotion interventions, thereby improving the subjective experience of exercise.

## Data Availability Statement

The raw data supporting the conclusions of this article will be made available by the authors, without undue reservation.

## Ethics Statement

The studies involving human participants were reviewed and approved by Ethics Committee of the Medical Faculty, University of Tübingen (# 882/2017BO1) and was registered at the German Clinical Trials Register (# DRKS00017446). The participants provided their written informed consent to participate in this study.

## Author Contributions

KD, FM, AN, AT, and GS conceptualized the paper. KD and FM performed the measurements and prepared the data. AN, AT, and GS supervised the study. PM contributed to the planning of the statistical procedures. KD and IR analyzed the data. KD wrote the first draft of the manuscript. FM provided the information for the procedures on the standardization of exercise intensity and IR for the model descriptions in the statistics section. All authors discussed and revised the manuscript before submission and read and approved the final manuscript.

## Conflict of Interest

The authors declare that the research was conducted in the absence of any commercial or financial relationships that could be construed as a potential conflict of interest.

## Publisher's Note

All claims expressed in this article are solely those of the authors and do not necessarily represent those of their affiliated organizations, or those of the publisher, the editors and the reviewers. Any product that may be evaluated in this article, or claim that may be made by its manufacturer, is not guaranteed or endorsed by the publisher.
