# Supplementary material for: Different Endurance Exercise Modalities, Different Affective Response: A Within-Subject Study
Source: Front Psychol. 2021 Aug 13;12:686661. doi: 10.3389/fpsyg.2021.686661 (PMC8411706; doi:10.3389/fpsyg.2021.686661)
Supplement: Supplementary file 1 [file Table_1.DOCX]

Supplementary Material

**Supplementary Table 1.** Control of modality-dependent influence of environment-related characteristics.

| Variable | F | p | | |
| --- | --- | --- | --- | --- |
|  |  |  | |  |
| ENVIRONMENT (ENV) | | | | |
| Intercept | 84.324 | | < .001*** | |
| Exercise Modality | 3.965 | | .020* | |
| ENV | 0.315 | | .575 | |
| **ENV x Exercise Modality** | **0.382** | | **.682** | |
|  |  | |  | |
| INTERACTION TRAINING SUPERVISOR (ITS) | | | | |
| Intercept | 37.344 | | < .001*** | |
| Exercise Modality | 0.202 | | .817 | |
| ITS | 0.643 | | .019* | |
| **ITS x Exercise Modality** | **1.059** | | **.152** | |

*Note.* The results represent associations of the environment (ENV) and the interaction with the training supervisor (ITS) on participants’ in-task (t_1_, t_2_) affective valence (Feeling Scale). Bold letters indicate the exercise modality-dependent association (*x* = interaction term). The impact of the training setting on the general affective state during exercise were operationalized using the following assessment: “The influences of the environment (e.g. temperature, space) were . . .” (ENV) and “The contact with training leaders was . . .” (ITS) with a rating scale from *very disturbing* (0) to *very conducive* (100).
**p* < .05. ****p* < .001 (Bonferroni adjusted).

**Supplementary Table 2.** Relative heart rate parameters in the three different exercise modalities.

|  | %HR_max_ | R-%HR_max_ | %HRR | R-%HRR |
| --- | --- | --- | --- | --- |
| MICE  (n = 210) | 68.87 (6.10)  53.84–83.82 | 60–70 | 50.01 (10.14) 20.62–72.74 | 45–55 |
| VICE  (n = 222) | 83.17 (6.64)  62.70–94.86 | 70–90 | 73.06 (10.99)  32.76–92.35 | 55–80 |
| HIIE  (n = 213) | 89.44 (4.34)  78.43–98.84 | > 90 | 82.81 (7.40)  61.28–98.16 | > 80 |

*Note.* Empirical means and ranges of relative heart rate measures compared to reference values (*R*) according to Binder et al. (2008) indicated that the participants were exercising at the intended intensity for each exercise modality. Only the mean value of %HRmax for HIIE was slightly below the targeted 90%. However, the %HRR value for HIIE was within the target range of over 80%. Standard deviations are presented in parentheses. %HR_max_ = percentage of maximum heart rate, %HRR = percentage of heart rate reserve, MICE = moderate-intensity continuous exercise, VICE = vigorous-intensity continuous exercise, and HIIE = high-intensity interval exercise.

**Supplementary Table 3.** Descriptive statistics of in-task affective valence in the three different exercise modalities.

|  | n | M | SD | SE | 95% CI | Range |
| --- | --- | --- | --- | --- | --- | --- |
| MICE | 232 | 2.92 | 1.31 | 0.09 | [2.75, 3.09] | -4.00–5.00 |
| VICE | 222 | 2.10 | 1.74 | 0.12 | [1.87, 2.33] | -5.00–5.00 |
| HIIE | 226 | 2.48 | 1.60 | 0.11 | [2.27, 2.69] | -5.00–5.00 |
| Total^a^ | 680 | 2.51 | 1.59 | 0.06 | [2.39, 2.63] | -5.00–5.00 |

*Note.* Raw data of affective valence (Feeling Scale) is presented without adjustment for the dependencies within clusters. MICE = moderate-intensity continuous exercise, VICE = vigorous-intensity continuous exercise, and HIIE = high-intensity interval exercise.
^a^40 participants: 3 sessions per exercise modality, two in-task time points (t_1_, t_2_) per session.
